# Effect of Oral Herbal Medicament on Scalp Seborrhea and Gastrointestinal Symptoms in a Male Patient: A Case Report

**Published:** 2018-07

**Authors:** Majid EMTIAZY, Elham ZAREIE, Laila SHIRBEIGI, Omid SADEGHPOUR, Parvin MANSOURI

**Affiliations:** 1. Dep. of Traditional Medicine, Faculty of Iranian Traditional Medicine, Shahid Sadoughi University of Medical Sciences, Ardakan, Yazd, Iran; 2. The Research Center of the Iranian Traditional Medicine, Shahid Sadoughi University of Medical Sciences, Yazd, Iran; 3. Dep. of Iranian Traditional Medicine, School of Traditional Medicine, Tehran University of Medical Sciences, Tehran, Iran; 4. Dept. of Herbal Medicine, Research Institute for Islamic & Complementary Medicine, Iran University of Medical Sciences, Tehran, Iran; 5. Skin and Stem Cell Research Center, Tehran University of Medical Sciences, Tehran, Iran

**Keywords:** Seborrhea, Traditional, Persian medicine, Functional dyspepsia, Triphala

## Abstract

A 32-yr-old man with a 10-yr history of scalp seborrhea referred to Skin and Stem Cell Research Center, Tehran, Iran, in 2015. He suffered from scalp seborrhea. Concurrent gastrointestinal symptoms and the changes in the clinical symptoms after consumption of the polyherbal traditional drug called Triphala are discussed. The scalp sebum was measured with a Sebumeter SM815. Gastrointestinal symptoms were followed using a valid questionnaire. After two months of treatment, scalp sebum secretion had decreased substantially. The patient also experienced remarkable improvement in gastrointestinal symptoms. Considering the positive effect of this known and safe polyherbal drug on skin sebum, it is an appropriate option for detailed large-scale clinical trials.

## Introduction

Seborrhea affects both men and women. It usually starts after puberty. The condition is not life-threatening but can predispose patient to acne and cause adverse psychological and social effects. Sebum secretion is a complex hormone-regulated condition influenced by age, genetics, nutrition and a hot and humid climate (1). The treatments recommended for seborrhea are mostly intended to treat complications such as acne and seborrheic dermatitis. These treatments might control a patient’s symptoms (2), but their negative side effects are evident (3).

Many dermatologic patients turn to complementary medicine and this has lead dermatologists to investigate the method (4). Traditional Persian medicine (TPM) texts suggest both topical and oral remedies for seborrhea (5). Triphala is the best known oral medication. It composed of three medicinal fruits: *Emblica officinalis* Gaertn. (Euphorbiaceae), *T. chebula* Retz. (Combretaceae), and *T. belerica* Retz. (Combretaceae). It is an antioxidant, antimutagenic, antimicrobial immunomodulator and also an enteroprotective drug. In addition, its effects on skin ulcers have been noted in studies. Triphala polyherbal medicament is safe for humans and animals (6).

The current case study is the first successful report of the use of Triphala to decrease sebum secretion and also follows changing procedure of gastrointestinal symptoms over the course of eight weeks of treatment.

## Case Report

A 32-yr-old man with a 10-yr history of scalp seborrhea referred to Skin and Stem Cell Research Center, Tehran, Iran, in 2015. He voluntarily participated in this study. He first was examined by a physician, who took a complete medical history and administered a dermatological examination. The patient had not used any topical or oral agents to reduce the oiliness of his skin for the previous 10 weeks. He had no symptoms of seborrheic dermatitis or any other dermatological disorder with the exception of acne lesions on his face and upper parts of the chest and back.

Informed consent was obtained directly and in writing from the patient before publication of this manuscript.

The patient also had been experiencing functional dyspepsia for the previous last three months as based on the Rome III criteria. He experienced postprandial fullness, excessive belching and upper abdominal bloating. His endoscopic and biopsy evaluations were normal. Omeprazole had been prescribed by a gastroenterologist for eight weeks but no improvement in gastrointestinal symptoms was observed.

**Fig. 1:**
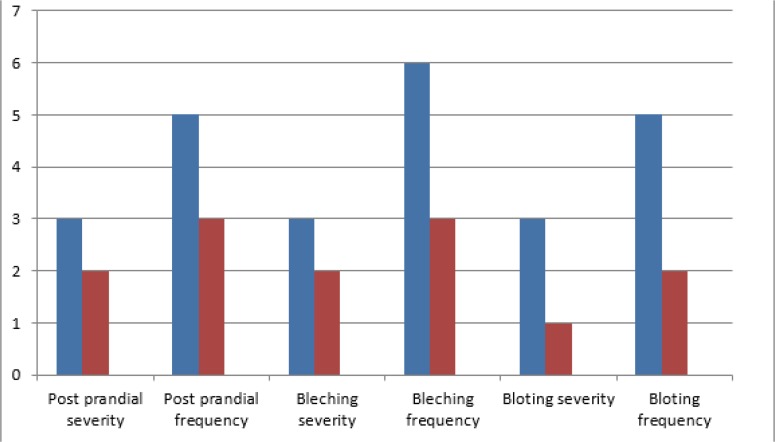
The severity and frequency of GI disorder symptoms assessed by Likert scale Severity: 0, absent, 1, mild (without influence on daily activities); 2, relevant (interfering with daily activities but not urging modification); 3, have influence in daily activities with urge modification) Frequency: 1, occurring < 1 d/mo; 2, occurring 1 d/mo; 3, occurring 2–3 d/mo; 4, occurring 1 d/wk; 5, occurring > 1 d/wk; 6, occurring every day

The scalp seborrhea was evaluated using a Sebumeter SM815. Sebumetry is a well-accepted method of measuring the casual levels of sebum. Sebumetry was performed on the patient’s vertex region 24 h after shampooing with his usual shampoo without a topical agent. The patient was also asked to record any changes in his GI symptoms in his self-report diary. The severity and frequency of the symptoms were assessed before and at the end of treatment using a Likert scale. During the 8-wk therapeutic period, the patient consumed capsules containing 500 mg of Triphala twice daily (after breakfast and dinner). No restrictions were placed on the frequency of shampooing. He was asked to inform his physician of any adverse effects. He also was visited two times during this period to examine for possible complications. No adverse effects or drug intolerance were detected during treatment. The treatment outcome showed a significant improving in his scalp seborrhea index, changing from 211 (μg cm^−2^) at the beginning of treatment to 67 (μg cm^−2^) at the end of 8 weeks of treatment. His gastrointestinal complications also improved dramatically noted in Fig. 1.

## Discussion

Proper functioning of the GI tract is a key to a healthy body from the perspective of TPM. The authors of TPM textbooks have described a strong relationship between GI dysfunction and seborrhea. An imbalance in the four senses of humor can cause stomach dysfunction (7). The presence of abnormal heat and moisture in the stomach can cause laxity in the stomach tissue that can weaken digestion and result in dyspepsia and introduced seborrhea as a clinical sign of GI dysfunction. Clinical experience had shown them that seborrhea can be managed by treating the underlying GI dysfunction. TPM physicians have recommended Triphala as an effective treatment to improve the symptoms of the GI disorder responsible for seborrhea (5).

Alternations in GI microbial communities are correlated with GI disorders including functional dyspepsia (8) as well as it may have a significant impact on skin disorders (9). Since Triphala may be able to modulate GI microbial ecology (10), its possible efficacy in a wide spectrum of GI and dermatologic disorders would not be out of mind.

## Conclusion

The current study reports the first documented case of the effect of Triphala on scalp seborrhea as well as its concurrent effects on functional dyspepsia. The mechanism of the treatment remains unknown; however, it could be a starting point for treatment of seborrhea after detailed clinical trials.

## Ethical considerations

Ethical issues (Including plagiarism, informed consent, misconduct, data fabrication and/or falsification, double publication and/or submission, redundancy, etc.) have been completely observed by the authors.
